# Microtechnology-based *in vitro* models: Mimicking liver function and pathophysiology

**DOI:** 10.1063/5.0061896

**Published:** 2021-10-15

**Authors:** Seung Yeon Lee, Donghyun Kim, Seung Hwan Lee, Jong Hwan Sung

**Affiliations:** 1 Department of Chemical Engineering, Hongik University, Seoul 04066, South Korea; 2 School of Electrical and Electronic Engineering, Yonsei University, Seoul 03722, South Korea; 3 Department of Bionano Engineering, Center for Bionano Intelligence Education and Research, Hanyang University, Ansan 15588, South Korea

## Abstract

The liver plays important roles in drug metabolism and homeostasis. The metabolism and biotransformation can not only affect the efficacy of drugs but also result in hepatotoxicity and drug-induced liver injury. Understanding the complex physiology of the liver and the pathogenetic mechanisms of liver diseases is essential for drug development. Conventional *in vitro* models have limitations in the ability to predict drug effects, due to the lack of physiological relevance. Recently, the liver-on-a-chip platform has been developed to reproduce the microarchitecture and *in vivo* environment of the liver. These efforts have improved the physiological relevance of the liver tissue used in the platform and have demonstrated its applicability to drug screening and disease models. In this review, we summarize the recent development of liver-on-a-chip models that closely mimic the *in vivo* liver environments and liver diseases.

## I. INTRODUCTION

The liver plays important roles in the metabolism of amino acids, carbohydrates, and nitrogen, as well as in detoxification, conjugation, and activation.^1,2^ In line with these roles, albumin, urea, and bile are secreted into the blood and intestine, and glucose is stored and generated in the liver.^3^ Moreover, toxins, drugs, and chemicals are transformed in the liver through xenobiotic metabolism, which comprises phase I and phase II metabolism.^2^ This metabolism and biotransformation of drugs affect the efficacy of drugs and can also cause hepatotoxicity and drug-induced liver injury, indicating the importance of accurate prediction of drug metabolic profile of both normal and diseased liver during drug development.^4,5^ The accuracy of prediction can be enhanced by understanding the complex physiology of the liver and the pathogenetic mechanisms of liver diseases. This understanding can be achieved when healthy and diseased liver models are constructed with improved physiological relevance. Animal models carry several limitations, such as ethical issues, differences between species, and extrapolation to humans.^6^ Conventional, cell-based *in vitro* models have limitations in the prediction of drug effects due to lack of physiological relevance.^7–9^

Recently, microtechnology has emerged and allowed the development of microchips that integrate cell culture models and microfluidics.^10^ Such approaches have been employed to mimic the *in vivo* environment with increased physiological relevance.^11,12^ Cells can be cultured in microenvironments with physiologically realistic environmental cues, such as cell-to-cell and cell-to-extracellular matrix (ECM) interactions, fluidic shear and mechanical stimuli, concentration gradient of oxygen and signaling molecules, leading to an improvement in the physiological relevance of cell behavior.^13^ The pursuit of such a concept of physiologically realistic *in vitro* models for the past two decades has resulted in the emergence of the organ-on-a-chip field, with particular focus on the construction of liver models.^14–16^ Furthermore, the liver models have been used in association with other organ models, where multi-organ chip models can recapitulate the complex interaction between different organs.^17,18^ Based on this initial development, recent studies have focused on liver disease models to simulate and understand the pathology of diseases.^2^

In this review, we summarize the recent progress on liver-on-a-chip models. First, we describe *in vitro* liver models that mimic the liver microenvironment. The improvement in hepatic and metabolic functions in these chip-based *in vitro* models can greatly increase their potential value as drug screening tools. Second, we summarize recent progress of gut–liver models, wherein liver models are connected to intestinal models to recapitulate the gut–liver crosstalk. Third, we describe recent progress of chip-based liver disease models. Liver disease models are important for understanding the mechanisms of disease development. Moreover, utilization of the appropriate disease model can provide better information regarding the efficacy of drugs. Finally, we propose future directions for the liver-on-a-chip models and the challenges of developing a better *in vitro* model system. In Secs. III and IV, we have selected and discussed highly cited pioneering works or recent representative research articles to understand the progress and highlight the improvement in the field of liver-on-a-chip.

## II. PHYSIOLOGY OF THE LIVER

### A. Structure and function of the liver

Histologically, the liver comprises several lobules arranged in the form of a hexagonal structure, which has a central vein and a hepatic portal triad at the corner.^19^ The hepatic portal triad consists of the hepatic portal veins, hepatic artery, and hepatic bile duct.^20^ The nutrient-rich and oxygenated blood supplied from the portal vein and hepatic artery mix and flow to the central vein through the hepatic sinusoid.

The hepatic sinusoid is the basic structural unit of the liver and comprises parenchymal and non-parenchymal cells.^21^ Parenchymal cells are hepatocytes arranged alongside the sinusoids. The apical side of hepatocytes is polarized with adjacent hepatocytes and forms tight junctions and bile canaliculi.^22^ The bile produced by hepatocytes flows into the bile duct through the bile canaliculi. The basal side of hepatocytes is bound to the space of Disse, which is located between the hepatocytes and sinusoids.^23^ Therefore, hepatocytes can be in contact with the blood through the fenestrae of the sinusoid.^24^ Non-parenchymal cells are composed of stellate cells, liver sinusoidal endothelial cells (LSECs), and Kupffer cells.^2^ LSECs form sinusoids and directly contact the blood as endothelial cells. LSECs have fenestration, which is different from the characteristics of vascular endothelial cells.^25^ Hepatic stellate cells are fibroblasts located in the space of Disse.^26^ The stellate cells help to maintain the morphology of LSECs and play a role in the deposition of the ECM upon activation in response to changing environment after infection and alcohol uptake.^2^ Kupffer cells are macrophages of the liver that are anchored to the LSECs in the lumen of liver sinusoids. These cells eliminate foreign particulates by phagocytosis and release various cytokines.^27^

The liver performs different functions, such as carbohydrate, lipid, and amino acid metabolism, ammonia clearance, urea synthesis, albumin and bile acid synthesis, and xenobiotic metabolism.^28^ Xenobiotic metabolism is particularly of interest in the drug development process to study drug toxicity. Metabolism is classified into phases I and II.^5^ Phase I metabolism is performed by cytochrome P450 (CYP 450), wherein drugs are modified by oxidation, reduction, hydrolysis, and dehydrogenation. Phase II metabolism involves sulfation or glucuronidation, which are performed by transferases.^29^ After phase II metabolism, the modified drugs can be excreted through the kidneys.

### B. Cells source for construction of a liver model

To construct *in vitro* liver models that resemble *in vivo* models, the source of hepatocytes is important. Hepatocyte sources include primary hepatocytes, hepatic cell lines, and stem cell-derived hepatocytes. Primary hepatocytes can be isolated from the liver of various species, such as mice, rats, and humans. They have been shown to preserve liver functions, including phase I and II enzyme activities. However, primary hepatocytes are difficult to subculture; these cells do not proliferate on dishes and wells and rapidly lose their hepatic functions. Moreover, the cost incurred is high, and the preserved functions differ depending on the batch.

Hepatic cell lines are derived from cancer cells. HepG2 cells obtained from human hepatocellular carcinoma can be stably cultured on a dish or plate. However, they have shown limitations in reproducing liver functions. For example, the expression and activity of CYP 450 are downregulated in cultured cells compared to that in primary hepatocytes. HepaRG cells are hepatic progenitor cells derived from the hepatocellular carcinoma of a patient infected with hepatitis virus.^30^ HepaRG cells can be subcultured and exhibit characteristics similar to those of primary hepatocytes through differentiation. Moreover, they can better reproduce liver functions as compared with HepG2 cells; however, compared to primary hepatocytes, the detection sensitivity of hepatotoxic drugs was lower.^31^

To construct a liver-on-a-chip model, one of the major hurdles is the limited supply of cell sources. Researchers have proposed the use of pluripotent stem cells (PSCs) to address this limitation. Alternatively, PSC-derived hepatocytes can be employed. These cells show stable liver functions and low variability between batches.^3^ However, their cultures can take a long time (>15 days) and require induction factors to mediate differentiation.^2^ Furthermore, metabolic activities of these cells were found to be lower than those of primary hepatocytes and HepaRG cells.^32^ The evolution of differentiation protocols is needed to substitute primary hepatocytes.

## III. LIVER MODELS

### A. *In vitro* models with liver zonation

Hepatic tissue can be divided into zones based on several factors, such as oxygen, and nutrients, and hepatocytes show different morphologies and functions depending on the zone.^33^ This is one of the specific characteristics of the liver known as the liver zonation. Among these factors, the oxygen gradient is one of the key factors affecting the metabolic functions of hepatocytes.^21,34–36^ The periportal areas of liver tissue are oxygen-rich zones, and hepatic functions, such as albumin and urea synthesis and glutathione-mediated detoxification, are relatively dominant. In the perivenous area, oxygen is relatively low and CYP enzyme activity is relatively higher.^37^ Despite the importance of hepatic zonation, the continuous model of such phenomena cannot be realized with conventional *in vitro* cell culture models. A microfluidic system is an ideal platform for achieving a stable laminar flow, owing to the low Reynold's number. Under laminar flow conditions, diffusion plays a major role in mass transfer, and the liver zonation can be simulated easily by letting the diffusion create the oxygen gradient. For example, Allen *et al.* co-cultured primary rat hepatocytes (PRH) and non-parenchymal cells in a perfusion bioreactor system.^38^ The authors introduced O_2_ through an inlet reservoir and monitored O_2_ concentration at the outlet by altering the various flow conditions. Under physiological oxygen gradient conditions, the cells showed a heterogeneous distribution of enzymes. In the oxygen-rich areas located upstream, phosphoenolpyruvate carboxykinase activity was dominant. In contrast, cytochrome P450 family 2 subfamily B (CYP450 2B) activity was dominant in the low-oxygen region located downstream. Cell death caused by the hepatotoxicity of acetaminophen was observed at the low-oxygen outlet.

**FIG. 1.**
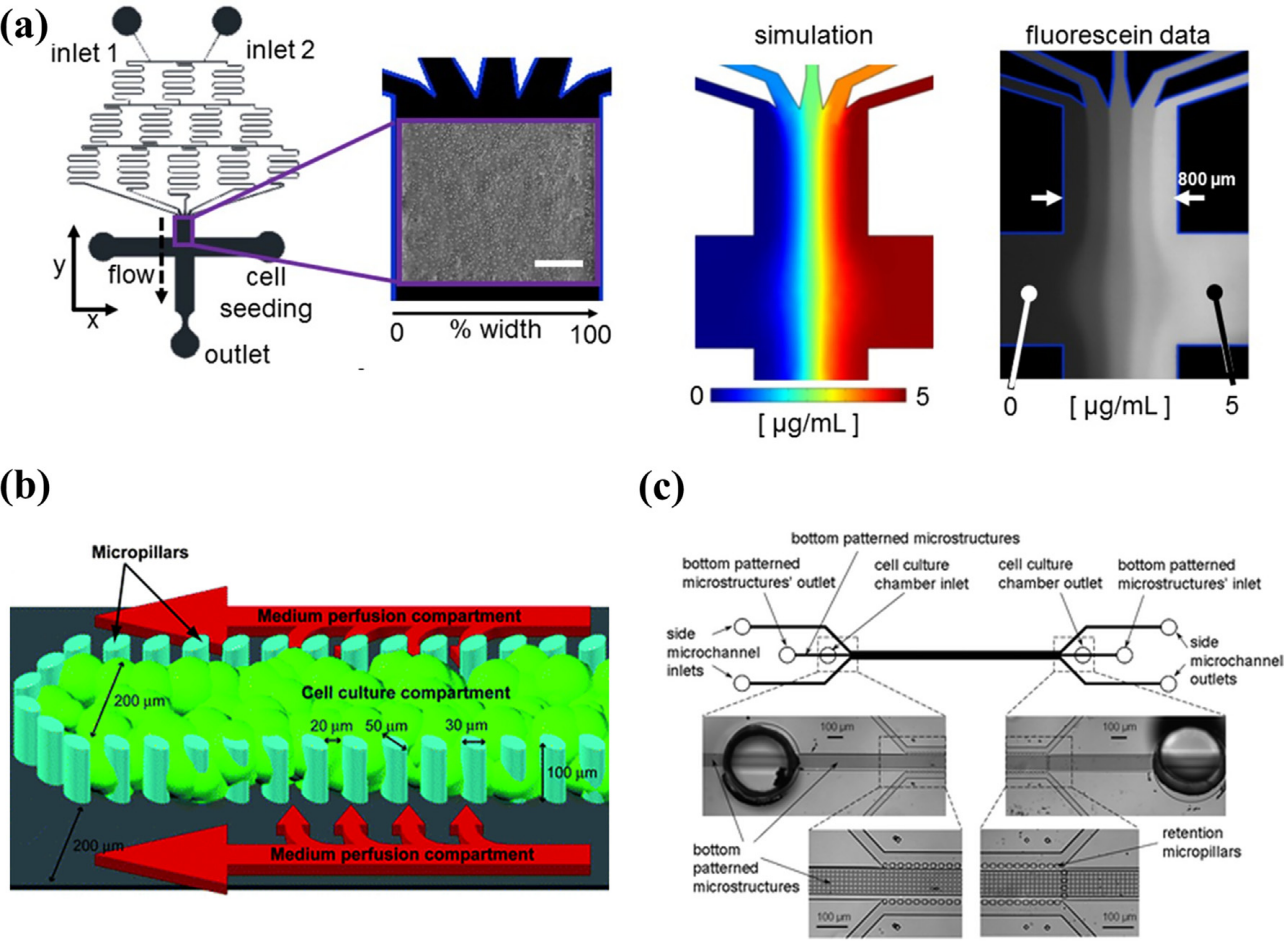
(a) Liver zonation feature-based models: A tree-like concentration gradient generator was combined with a cell culture chamber (Bar: 200 *μ*m). Reproduced with permission from McCarty *et al.*, Sci. Rep. **6**, 26868 (2016). Copyright 2016 Author(s), licensed under a Creative Commons Attribution (CC BY) license.^39^ (b) Structural feature-based models: endothelial barrier was integrated into the micropillar form of microfluidic device to mimic the liver sinusoid. Reproduced with permission from Toh *et al.*, Lab Chip **9**, 2026 (2009). Copyright 2009 The Royal Society of Chemistry.^47^ (c) Structural feature-based models: a microstructure was added to the bottom of the cell culture chamber. Hepatocytes were surrounded by the cell culture medium. Reproduced with permission from Goral *et al.*, Lab Chip **10**, 3380 (2010). Copyright 2010 The Royal Society of Chemistry.^48^

In several studies, zonation was created using a tree-like microfluidic gradient generator and liver cells were subjected to the created zonation. Usta and co-workers demonstrated zonation-dependent carbohydrate and nitrogen metabolism.^1,39^ The authors applied concentration gradients of insulin and glucagon to primary rat or human hepatocytes, and glycogen storage and urea synthesis were observed using periodic acid-Schiff staining and carbamoyl phosphatase synthetase I staining [Fig. 1(a)]. Glucose release and urea formation were predominant in zone 1 (periphery areas). Moreover, the gradient of 3-methylcholanthrene provided predominant alcohol degradation and acetaminophen toxicity in the 3-methylcholanthrene rich zone. These examples of recapitulating the liver zonation by controlling the oxygen gradient with microfluidic technique are a typical example where microfluidics can help to recreate the *in vivo* tissue environment.

### B. *In vitro* models with shear stress

The liver is a vascular organ where 25%–30% of the total blood entering from the hepatic artery and portal vein passes through the sinusoid and flows out through the central vein.^40^ Therefore, hepatocytes and non-parenchymal cells experience shear stress due to blood flow. Shear stress is a mechanical stimulus that cannot be reproduced in conventional *in vitro* systems, such as well plates or dish cultures. After the development of liver-on-a-chip system, hepatocytes have been cultured under dynamic conditions through the application of flow and shear stress. Several studies have investigated the effect of shear stress on functions of hepatic cells.

Tanaka *et al.* designed a microchip and applied shear stress (1.4 to 60 dyne/cm^2^) to HepG2 cells.^41^ These authors proved that the flow could provide oxygen and nutrients to the cells. However, high flow rate damaged the cells, owing to shear stress. Vinci *et al.* cultured primary hepatocytes in a multi-chamber modular bioreactor (flow, 250–500 *μ*L/min; shear stress, 5 × 10^−6 ^dyne/cm^2^) and compared CYP 450 enzyme activities and biological parameters under static conditions for 7–21 days.^42^ The mRNA expression of detoxification- or xenosensor-related genes was upregulated in 2-week cultures under dynamic conditions. Rashidi *et al.* applied shear stress (2.9–4.7 × 10^−5^ dynes/cm^2^) to hepatocyte-like cells derived from human embryonic stem cells and induced PSCs.^43^ Under fluid shear stress conditions, the phenotype of hepatocyte-like cells improved and CYP1A2 activity enhanced by fivefold as compared with that observed in static cultures. Furthermore, secretion of alpha-fetoprotein, a fetal marker, decreased by fourfold compared to that observed under static conditions.

These investigations suggest that most livers-on-a-chip apply shear stress to cells. Although the applied shear stress differs depending on the model under study, the induced shear stress allows improvement of various aspects of hepatic cells, such as long-term cultures and production. Therefore, the optimized shear stress should be studied depending on the system. The applied shear stress in the liver-on-a-chip is lower than the reported value at the sinusoid (0.1–0.5 dynes/cm^2^).^44^ These differences may be caused by the presence of sinusoid endothelial cells and the space of Disse. Therefore, the relationship between shear stress and the effect of non-parenchymal cells or topology of sinusoids should be further studied to construct a liver-on-a-chip that mimics the *in vivo* environment.

### C. *In vitro* models recapitulating the structural features

Several researchers have integrated an endothelial barrier into the microfluidic device to mimic the liver sinusoid. The unique structure of the hepatic sinusoid has been described in Sec. III B. In the hepatic sinusoid, hepatocytes are supplied with nutrients and oxygen through numerous sinusoid capillaries. However, conventional culture systems may fail to reproduce these supplement conditions. Several studies have been conducted to overcome these limitations. In these studies, pillars that recapitulate an endothelial barrier were developed using microtechnology. Liver cells were cultured inside these pillars, and cell culture medium was supplied through the outside of pillars. Therefore, oxygen and nutrients can be supplied to liver cells through gaps between pillars. Hepatic functions of liver cells can be improved through these perfusion culture systems.

First, Lee *et al.* designed an endothelial-like barrier (2 *μ*m in width, 1 *μ*m in height, and 30 *μ*m in length) that surrounded the cell culture region (50 *μ*m in width, 30 *μ*m in height, and 500 *μ*m in length).^45^ After primary hepatocytes were introduced into the cell culture region, the cell culture medium was provided through a convective transport. The cell culture medium was allowed to diffuse into the endothelial-like barrier from the vessel. Under these conditions, the viability of primary rat and human hepatocytes was maintained for more than 7 days. The authors provided diclofenac to cells to examine the occurrence of metabolism-mediated hepatocyte toxicity. When the hepatocytes were exposed to diclofenac for 4 h, viability was preserved. However, exposure of hepatocytes to diclofenac for 24 h caused cell death, indicating the toxicity of the drug. This is a pioneering work that integrates an endothelial-like barrier and reproduces the blood flow in a sinusoidal structure. However, hepatocytes were cultured in 2D environments.

Toh *et al.* improved the system of Lee *et al.* to incorporate three-dimensional (3D) culture of cells.^46^ The height of the cell culture region increased to 100 *μ*m, which was surrounded by elliptical micropillar arrays to prevent clogging of seeded cells and supply cell culture medium. After the cells were seeded into the cell culture region, a 3D ECM was formed by injection of methylated collagen and terpolymer hydroxylethylmethacrylate–methylmethacrylate–methylacrylic acid. Therefore, the establishment of cell-to-cell and cell-to ECM interactions can be simulated by the system. The authors cultured various cells, including HepG2 and primary hepatocytes to demonstrate the versatility of the system. In the following study, primary hepatocytes were cultured on the chip [Fig. 1(b)].^47^ The functions of hepatocytes were demonstrated via albumin production and exhibition of phase I and II metabolic activities. The authors examined the concentration-dependent hepatotoxicity of five model drugs using a concentration gradient system. Authors found that the IC_50_ values of the model drugs correlated with those of the *in vivo* lethal dose 50 (LD_50_) in rats.

In another related study, Goral *et al.* added a microstructure to the bottom of the cell culture chamber designed by Toh *et al.* [Fig. 1(c)].^48^ As hepatocytes were surrounded by the cell culture medium, virtual suspension culture was realized, and the interactions between the cell and surface were minimized. The viability of hepatocytes was maintained for two weeks. The cells formed a 3D tissue-like structure without the addition of biological or synthetic matrices or coagulants. In the developed chip, the polarity of hepatocytes, formation of bile canalicular structures, and transport function of metabolites were demonstrated using multidrug resistant protein 2, which is important for the efflux of drug metabolites. Additionally, the formation of gap junctions was observed via the expression of connexin 32. In this study, additional supplementation of the medium through the bottom microchannel improved the phenotype and function of liver cells without matrices. Thus, a sufficient supply of medium is imperative for liver cell cultures.

Mimicking the 3D structure of the liver tissue could enhance the liver-specific functions of cell lines and human-induced PSC-derived hepatocytes. Banaeiyan *et al.* designed liver-lobule-like hexagonal tissue culture chambers that contained flow channels mimicking the blood flow in the liver tissue.^49^ In the developed chip, HepG2 cells were cultured for 14 days and human-induced pluripotent stem cell (hiPSC)-derived hepatocytes were cultured for 21 days. Secretion of albumin and synthesis of urea were demonstrated. The formation of bile canaliculi was observed by performing 5-and-6-carboxy-2',7'-dichlorofluorescein diacetate (CDFDA) staining. These studies demonstrate that the recapitulation of structural features surrounding the hepatocytes, such as the sinusoids and the lobule structure, has important implications in eliciting realistic responses from the cells.

### D. Co-culture models using non-parenchymal cells

The liver consists of parenchymal and non-parenchymal cells. The parenchymal cells comprise 80% of the liver mass and consist of hepatocytes, while non-parenchymal cells comprise 20% of the liver mass and consist of liver sinusoidal endothelial cells, hepatic stellate cells, and Kupffer cells.^50^ Although the non-parenchymal cells occupy a small portion of the liver, these cells are important for establishing the crosstalk between hepatocytes and control cellular functions.^51,52^ Several studies have focused on the co-culture of non-parenchymal cells with hepatocytes in a microfluidic system.

Shuler group co-cultured primary human hepatocytes and non-parenchymal cells under gravity-based flow conditions.^53^ The system consisted of two polydimethylsiloxane (PDMS) layers, and each layer contained a microchannel. The microchannels were separated using a polycarbonate membrane. Primary human hepatocytes and non-parenchymal cells were co-cultured on the 3D scaffold and integrated on the membrane. Gravity-based flow was induced by using a rocking platform. Under gravity-based flow conditions, albumin and urea syntheses were enhanced compared to those under static conditions. The activity of CYP 1A1 and CYP 3A4 did not differ between the flow and static conditions. The authors examined the response of non-parenchymal cells to bacterial lipopolysaccharide (LPS), and the production of interleukin 8 (IL-8) was demonstrated for one week. Although the authors have demonstrated a co-culture system of parenchymal and non-parenchymal cells, the cells were mixed in a 3D scaffold, and the spatial arrangement was not reproduced. To overcome this limitation, several studies have attempted layer-by-layer cultures of parenchymal and non-parenchymal cells.

**FIG. 2.**
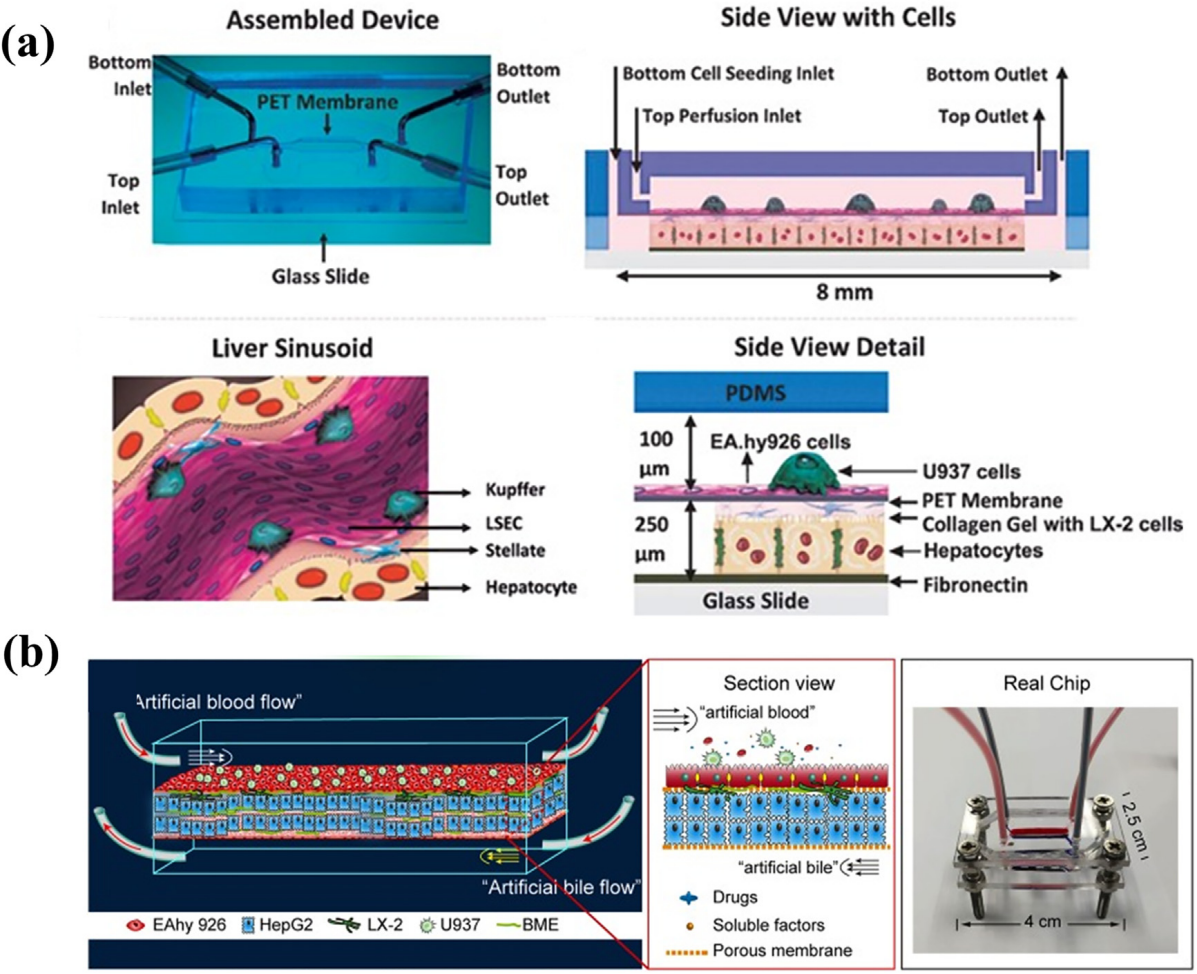
Co-culture models using non-parenchymal cells: The hepatocytes were co-cultured with liver sinusoidal endothelial cells, hepatic stellate cells, and Kupffer cells. (a) The system separated two layers by membrane. Reproduced with permission from Prodanov *et al.*, Biotechnol. Bioeng. **113**, 241 (2016). Copyright 2016 John Wiley and Sons.^54^ (b) The system separated three layers by membrane. One layer was used for artificial bile layer. Reprinted with permission from Deng *et al.*, Biomicrofluidics **13**, 024101 (2019). Copyright 2019 AIP Publishing.^56^

Prodanov *et al.* designed two PDMS layers containing microfluidic channels [Fig. 2(a)].^54^ The microfluidic channels were separated using a polyethylene terephthalate membrane. Primary hepatocytes and LX-2 cells (human hepatic stellate cell line) were seeded in the bottom channel. EAhy926 cells (human umbilical vein cell line; EAhy926 cells represent sinusoidal endothelial cells) and U937 cells (pro-monocytic, human histiocytic lymphoma cell line; U937 cells represent Kupffer cells) were seeded in the top channel. The cell culture medium was provided to the top channel. The co-culture was maintained for 28 days, and polarization of hepatocytes and formation of bile canalicular network were observed. Under flow conditions, albumin and urea syntheses were higher than those observed under static conditions. There was no difference in CYP3A4 activity observed between the static and flow conditions. In this study, non-parenchymal cell lines (LX-2, U937, and EAhy926) were co-cultured with hepatocytes to demonstrate long-term cultures under flow conditions.

In addition, lipopolysaccharide (LPS) infections were simulated using a co-culture system. Du *et al.* isolated four types of primary mouse hepatic cells (hepatocytes, stellate cells, sinusoidal endothelial cells, and Kupffer cells) and applied them to the microfluidic system.^55^ Similar to the design reported by Prodanov, the chip consisted of top and bottom channels. The channels were separated using a polyester membrane. The hepatocytes and stellate cells were cultured in the bottom channel, whereas endothelial cells and Kupffer cells were cultured on the top channel. The cell culture medium was allowed to flow through the top channel. In the presence of shear flow, the levels of the hepatocyte growth factor that were only secreted by non-parenchymal cells were increased, and the activities of CYP 1A2 and CYP 2D6 showed enhancement. When four types of hepatic cells were co-cultured, the recruitment of neutrophils was occurred, and the adhesion of neutrophils was higher than that observed with the monoculture of liver sinusoidal endothelial cells or the co-culture of liver sinusoid epithelial cells and Kupffer cells.

Furthermore, the chip design was improved by including the bile flow to address the cholestasis issues previously reported for liver chips. Lin *et al.* designed an artificial liver blood flow and artificial bile flow. The chip consisted of three layers separated by two polycarbonate membranes [Fig. 2(b)].^56^ EAhy926 cells were cultured on the upper side of the top porous membrane, and LX-2 cells were cultured on the lower side of the top porous membrane. HepG2 cells were mixed with a basement membrane extractant gel and loaded into the middle layer. The channels of the top and bottom layers were used for enabling artificial liver blood flow and artificial bile flow, respectively. Polarization of HepG2 cells and formation of canaliculus-like structures were observed by performing CDFDA staining. Active transportation was demonstrated using a bile acid analogue, cholineyl-lysyl-fluorescein. Additionally, hepatic functions such as albumin and urea syntheses and phase I and phase II metabolic activities of HepG2 cells were superior to those observed with well plate, monoculture, and static culture models. The cells cultivated using this system showed higher sensitivity in terms of hepatotoxicity than those cultivated using well plate-based static cultures when treated with hepatotoxicity-inducing drugs.

Recently, Ingber group used their microfluidic liver chip to reproduce human and cross-species drug toxicities.^57^ The chip comprised of an upper parenchymal channel and a lower vascular channel, with the channels separated by a porous membrane. The rat, dog, and human primary hepatocytes were cultured in the upper channel, while liver sinusoidal endothelial cells, Kupffer cells, and hepatic stellate cells were cultured on the porous membrane of the lower channel. Species-specific drug toxicities alongside species-specific differences in response to the drugs were simulated between humans and animals, through a diverse range of phenotypes, including hepatotoxicity, modeling of steatosis, and fibrosis. These approaches allow for the prediction of liver toxicity and provide information about the relevance of drug-induced liver toxicities between humans and animals. Species-specific drug toxicities cause failure of the drug development process. This work shows that the liver-on-a-chip has potential for the early prediction of drug efficacy and minimizes the late-stage failure of drug development caused by differences in species.

In addition, iPSC-derived hepatocytes (iHep) were co-cultured with non-parenchymal cells. Bircsak *et al.* applied microfluidic liver chip for high throughput hepatotoxicity screening.^58^ The authors used a OrganoPlate from Mimetas alongside an automated liquid handling robot. The microfluidic liver chip consisted of two microfluidic channels for an organ and a blood vessel. In the organ channel, aggregates of iPSC-derived hepatocytes (iHep) were cultured with the ECM, while the vascular channel consisted of endothelial cells (HMEC-1) and Kupffer-like immune cells (THP-1). The hepatic functions were maintained for 15 days. Furthermore, 159 compounds with known hepatotoxicity were tested for hepatotoxicity to show that the automated systems allowed high throughput screening of hepatotoxic compounds. iPSC cultures and application of the automated system show that the liver-on-a-chip system can be potentially applied for personalized medicine and drug screening.

Although the design of the microfluidic systems and the type of cells were different, the co-culture of hepatocytes with non-parenchymal cells under flow conditions resulted in improved hepatic functions that resembled the *in vivo* hepatic functions more accurately. Based on these studies, co-culture with flow should be considered as an essential element in the construction of physiologically relevant liver-on-a-chip systems.

### E. Three-dimensional (3D) cluster (spheroid or organoid)-based models

Several researchers have focused on the 3D cell-to-cell and cell-to-ECM interactions *in vivo*.^59^ Although 2D culture models of liver cells have been conventionally used for pharmacological purposes, the models have shown limitations in simulating 3D interactions. To overcome these limitations, 3D cluster models based on spheroids and organoids have been developed using hepatocytes or iPSC.^60^ The 3D cluster models improved liver-specific functions of 2D models because 3D interactions of the *in vivo* environment were more precisely recapitulated.^61^

Efficient formation of 3D clusters has been recently achieved through the fabrication of scaffolds using microtechnology. The scaffold was combined with a microfluidic system-based perfusion culture system. Griffith and co-workers fabricated a bioreactor that integrate scaffolds.^62^ The cell culture medium was injected into the inlet of the bioreactor and perfused through the scaffolds to the outlet of the bioreactor. A single-cell suspension and pre-aggregated spheroids of primary hepatocytes were cultured for two weeks. Pre-aggregated spheroids maintained their 3D tissue-like structure for 2 weeks, and single-cell suspension loss was observed after 1 week. Based on the study, the authors expanded their system to a multiwall plate-based bioreactor for conducting high-throughput culture.^63^

**FIG. 3.**
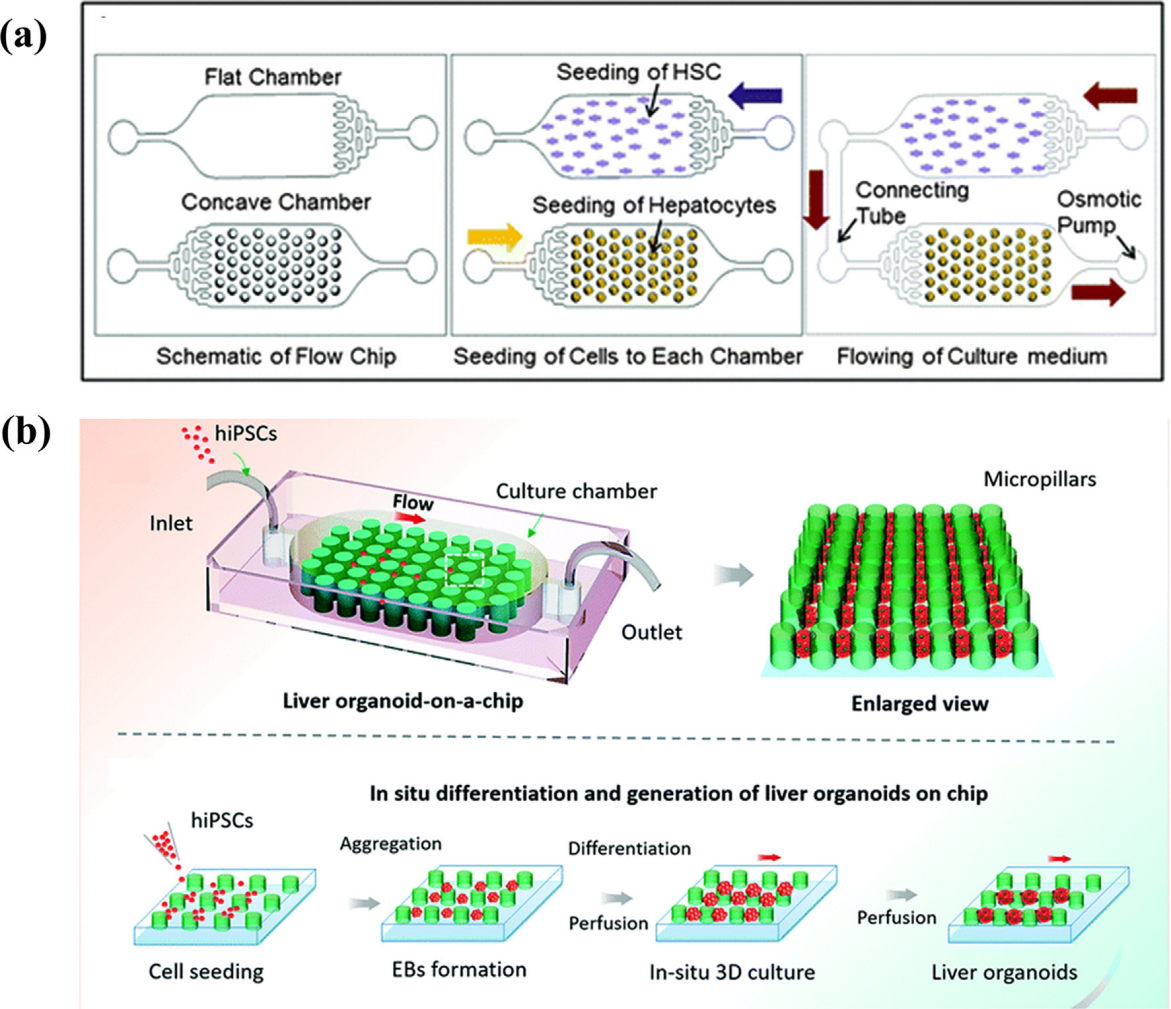
3D cluster (spheroid or organoid)-based models: (a) primary hepatocytes and hepatic stellate cell-based spheroids were connected with an osmotic pumping-based microfluidic system and communicated without direct cell-to-cell contact. Reproduced with permission from Lee *et al.*, Lab Chip **13**, 3529 (2013). Copyright 2013 The Royal Society of Chemistry.^65^ (b) Liver organoid-on-a-chip system: *in situ* differentiation and generation of liver organoids from hiPSCs was enable in a perfusion-based micropillar chip. Reproduced with permission from Wang *et al.*, Lab Chip **18**, 3606 (2018). Copyright 2018 The Royal Society of Chemistry.^69^

Several studies have focused on the interaction between parenchymal and non-parenchymal cells in 3D cluster structures. Lee *et al.* fabricated a concave microwell array using PDMS. Lee *et al.* fabricated a concave microwell array using PDMS.^64^ The concave microwell array facilitated the formation of uniform-sized spheroids as compared to plane surface and cylindrical microwells. The authors demonstrated that albumin secretion by primary hepatocytes and hepatic stellate cell-based heterospheres was 1.2-fold higher than that of primary hepatocyte-based hepatospheres. In the following study, the concave wells were combined with an osmotic pumping-based microfluidic system [Fig. 3(a)].^65^ The flow contributed to the formation of spheroids, long-term maintenance, and establishment of cell communication between primary hepatocytes and hepatic stellate cell-based spheroids without the necessity of direct cell-to-cell contact. In comparison with hepatocyte mono-cultured spheroids, primary hepatocytes and hepatic stellate cells in contact with the flow showed a twofold increase in albumin secretion and a 1.5-fold increase in urea synthesis. In this work, the microfluidic system was used for communication between parenchymal and non-parenchymal cells and revealed the importance of communication between cells.

Several research groups have attempted to encapsulate spheroids into hydrogels. The encapsulation of spheroids allows maintenance of functionality during long-term cultures.^66^ Bhise *et al.* developed a platform for long-term culture of HepG2/C3A spheroids through encapsulation of spheroids into the gelatin methacryloyl hydrogel and perfusion-based culture.^67^ HepG2/C3A spheroids formation was achieved using a PDMS microwell, and spheroids were mixed with the hydrogel. Then, the mixture was printed on the chamber by 3D printing and crosslinked via UV exposure. The spheroids demonstrated hepatic function for 30 days. Expression of cytoplasmic filament protein, cytokeratin 18, tight junction protein ZO1, biliary canalicular transporter protein, and MRP2 was observed after 30 days of culture.

Bhatia *et al.* encapsulated aggregated primary human hepatocytes and 3T3-J2 murine fibroblasts into PEG-DA using a droplet microfluidic system.^68^ The encapsulated cells were then trapped by using C-shaped traps and were cultured under perfusion conditions for 28 days. In general, primary hepatocytes rapidly lose their viability and functionality. However, the combination of encapsulated spheroid and perfusion-culture systems could be a solution to overcome the limitations of primary hepatocytes.

Organoids derived from PSCs show the characteristics of native organs and have also been combined with a microfluidic system. Wang *et al.* designed a microfluidic chip that contained a micropillar array structure [Fig. 3(b)].^69^ After seeding human iPSCs, long-term 3D cultures, and embryoid bodies (EBs), *in situ* hepatic differentiation and liver organoids were observed in the chip under perfusion conditions. Liver organoids showed heterogeneity through growth and differentiation of hepatocytes and cholangiocytes. This work demonstrates the potential of combination 3D cultures of human hepatic organoids and a perfusion culture system in organoid-based constructions of a liver-on-a-chip model.

Various methods for the formation of the 3D clusters have been introduced, and such clusters have shown improved hepatic functions. Furthermore, combination with the perfusion-based models and incorporation of clusters into hydrogels facilitated the formation of an ECM environment and enabled long-term culture with enhanced hepatic functions.

### F. Multi-organ models for simulation of gut–liver interactions

After the administered foods and drugs are pass through the small intestine, they are transferred to the liver through portal veins and metabolized by phase I and II metabolism. These processes are referred to as first-pass metabolism and are important in determining the effects of drugs and understand action mechanism of drugs. In several studies, the intestine compartment and liver compartment were connected by microchannel to simulate the first-pass metabolism.^70^ The ingredients of foods and drugs were crossed and metabolized in the intestinal compartment and delivered to the liver through microchannels. Then, intestinal metabolites undergo liver metabolism. Therefore, these intestine and liver interactions could simulate the effect of foods and drugs on the other organs.

Precision-cut slices were used to simulate the gut–liver interactions. As precision-cut slices can be obtained from animals by surgical methods, they retain the features of organs. Groothuis and co-workers integrated intestine and liver precision-cut slices obtained from rats into microfluidic chambers to demonstrate gut–liver communication.^71^ First-pass metabolism was mimicked by transferring metabolites in intestinal slices to the liver slices using connected flow. The bile acid, chenodeoxycholic acid, induced the expression of fibroblast growth factor 15 in the intestinal compartment, which resulted in a further decrease in the expression of CYP7A1 in the liver compartment. Although the physiological relevance of the slice was high, the supplement of the slice is limited since it should be extracted from animals. Given this limitation, cells have been used to reproduce the gut–liver interactions.

In many studies, Caco-2 (human colorectal adenocarcinoma cells) and HepG2 cells were cultured in the gut and liver compartments, and drug metabolism was studied based on the constructed system. Choi *et al.* compared the monoculture and co-culture of Caco-2 and HepG2 cells and demonstrated that CYP enzyme activity was higher in the co-culture system than that observed in the monoculture system.^72^ Leclerc and co-workers designed polycarbonate cell culture inserts and integrated them into a microfluidic chip, where Caco-2 TC7 and HepG2 C3A cells were cultured.^73^ The authors simulated the absorption of phenacetin by Caco-2 cells, and the transport and metabolism of phenacetin to acetaminophen by HepG2 C3A cells.

Several studies have focused on the effects of metabolized drugs on other organs and cancer cells for drug screening. In conventional culture systems, target cells, such as cancer cells, are treated with drugs. However, drugs are metabolized through the gut and liver, and their effects may differ. Therefore, consideration of first-pass metabolism is essential for predicting the efficacy of drugs. Sato and co-workers added MCF-7 cells to Caco-2 and HepG2 cells to establish a cancer model.^74^ The authors injected cyclophosphamide (CPA), epirubicin (EPI), 17-β estradiol (E2), or soy isoflavone (IF) into the system as model drugs. CPA addition decreased the viability of MCF-7 cells by metabolites produced by the metabolism of HepG2 cells. In contrast, the effects of E2 and IF decreased with metabolism of HepG2 cells. In a subsequent study, the authors examined the effect of CPA and tegafur (TGF) in the presence of gastric juices.^75^ CPA retained its anticancer activity, whereas TGF was degraded by gastric juices and lost its activity. These results were consistent with the characteristics of model drugs and indicated that the multi-organ model could predict the drug effects more accurately than the conventional monoculture system.

Shuler group designed an *in vitro* microscale cell culture analog (μCCA) system.^76^ The system consisted of the gastrointestinal (GI) tract, liver, and lung compartments. Caco-2, HepG2, and L2 cells were cultured in each compartment. The injection of acetaminophen into the Caco-2-layer inflicted damage on HepG2 or L2 cells through metabolism and depletion of glutathione in liver cells. The authors added mucus-secreting cells (HT29-MTX) and artificial chyme to accurately simulate the effect of orally administered drugs.^77^ In a subsequent study, the toxicity of nanoparticles was demonstrated using μCCA.^78^ The nanoparticles induced changes in the integrity of liver cells and released aspartate aminotransferase (AST). The Shuler group considered the drug pharmacokinetics (PK) and pharmacodynamics (PD) as well as the gut–liver interactions while designing the μCCA. This provides more accurate information about the efficacy of drugs because the drug absorption and metabolism closely resemble those under *in vivo* conditions.

**FIG. 4.**
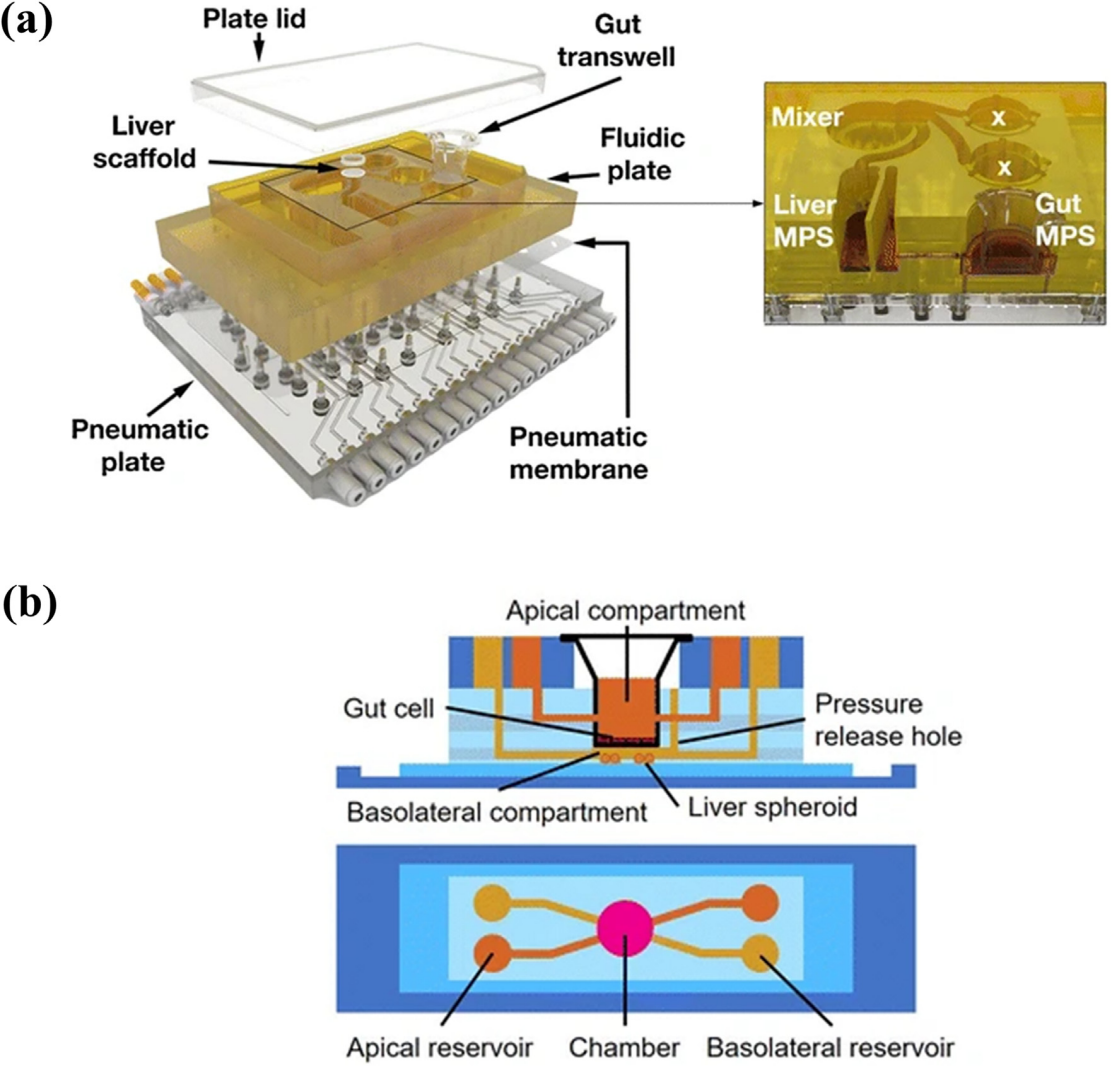
Multi-organ models for simulation of gut–liver interactions: gut and liver compartments were integrated in a single chip. (a) Cell culture medium was circulated by integrated valve and pump. Tsamandouras *et al.*, AAPS J. **19**, 1499 (2017). Copyright 2017 Author(s), licensed under a Creative Commons Attribution (CC BY) license.^80^ (b) Cell culture medium was circulated by gravity-induced flow. Reprinted with permission from Lee *et al.*, Biomed. Microdevices **19**, 100 (2017). Copyright 2017 Springer Nature.^83^

Several studies have focused on the fluid-to-tissue ratio to simulate *in vivo* systems more accurately. In many studies, a peristaltic pump was used to circulate cell culture medium. Thus, higher volumes of cell culture medium were used compared with the fluid-to-tissue ratio observed *in vivo*. This may cause dilution of metabolized drugs and make the analysis difficult. To solve this problem, an on-chip peristaltic pump was integrated into the system to minimize the liquid volume.^79^ Human primary intestinal epithelial cells were cultured in a transwell insert and combined with the intestinal compartment of the system. Liver spheroids were connected to the intestinal compartment. The repeated dose of troglitazone was simulated, and the response was examined using mRNA expression profile and immunohistochemical analyses. Griffith group combined gut transwell with a previously designed system that integrated scaffolds for the culture of liver cells [Fig. 4(a)].^80^ Authors reported that the pharmacokinetics of diclofenac and hydrocortisone were also simulated. Kimura and co-workers applied hiPS cell-derived intestinal cells and fresh human hepatocytes, which were isolated from PXB mice to pneumatic-pressure-driven system.^81^ The co-culture of hiPS-intestinal cells and PXB cells maintained the function of hiPS-intestinal cells and enhanced albumin production, metabolic function, and liver-specific gene expression of the PXB cells rather than those of the monoculture. These approaches can resolve fluid-to-tissue issues. However, the fabrication of integrated pumps and operation is difficult for non-experts and is one of the obstacles in the use or commercialization of the liver-on-a-chip system. Therefore, a more user-friendly system, such as a pumpless system, is warranted.

As another approach, several researchers have proposed the benefit of gravity flow-based, pumpless system. Sung and co-workers developed a gut–liver chip consisting of two separate layers for the gut (Caco-2) and liver (HepG2) cells.^82^ The gut and liver cells were cultured in a single chip using a gravity flow machine. The co-culture enhanced the metabolic activity of CYP enzymes and metabolic profile of the flavonoid apigenin was similar to the reported profile. In a subsequent study, Caco-2 cells were cultured on a 3D villi scaffold in the gut compartment, whereas liver spheroids were cultured in the liver compartment [Fig. 4(b)].^83^ The 3D culture provides a more accurate PK model of paracetamol compared with the 2D model, as the absorption surface area was larger and metabolic capacity was higher. In this work, a pumpless system using gravity flow was applied. However, the circulation of cell culture medium is different *in vivo*. *In vivo*, the absorbed food or drug moves to the liver where it is metabolized. Metabolites are then distributed to the body through blood circulation. Therefore, the gut–liver interactions are unidirectional.

Shuler's group developed a modular pumpless system for the co-culture of gut and liver cells.^84^ After the gut and liver cells were cultured in the separate module, the modules were combined for co-culture. The gravity flow-based system can provide a fluid-to-tissue ratio similar to that observed *in vivo*. Additionally, the use of a passive valve provided unidirectional flow and close simulation of sequential interactions between the gut and liver.^85^

The gut–liver interactions are important for the prediction of drug efficacy. Several studies have been conducted to study interactions between the gut and liver; many researchers have attempted to simulate these interactions more closely to *in vivo* conditions. In this section, we summarize the gut–liver on-a-chip system, which has the potential for prediction of drug efficacy.

## IV. LIVER DISEASE MODELS

Liver disease is one of the largest causes of death worldwide.^86^ Causes of liver disease include viruses, obesity, and alcohol. As the standard of living and average life expectancy increase, the prevalence of metabolic liver diseases, such as nonalcoholic fatty liver and alcoholic fatty liver, increases, and the prevalence of end-stage liver diseases, such as liver failure, cirrhosis, and liver cancer, is also increasing. Therefore, there is a need for a liver disease model for understanding liver physiology and pathophysiology and evaluating effective therapeutic agents.^87^

Liver disease models can be largely categorized as *in vivo* and *in vitro* models. The *in vivo* model is an animal model and plays an important role in the physiology of liver disease, target validation, and evaluation of new therapeutic agents.^87^ However, animal models incur ethical issues, and it is still difficult to interpret human diseases and develop therapeutics owing to differences in pathophysiology between humans and animals.^88^ The *in vitro* models generally use cells in a 2D form in a dish or well and has the advantage of relatively convenient experimental method. However, the existing 2D cell culture system has a limitation in its physiological relevance, because it cannot realize the physiological structure and microenvironment of the liver. Therefore, it is necessary to develop a model that overcomes the limitations of existing liver disease models.^89–91^

Recent studies on liver disease models have made various attempts to overcome the limitations of existing liver disease models. One of those attempts is liver-on-a-chip. The liver-on-a-chip system can not only reproduce the flow in the body but also reproduce the structure of the liver through structural design. In addition, 3D tissue culture, such as spheroid, organoid, and iPSC or co-culture with non-parenchymal cells, can simulate the physiological structure of hepatocytes and blood vessels.^92^ Here, we introduce various liver disease models using liver-on-a-chip, categorized by different diseases.

### A. Inflammation

Liver inflammation is a major factor in liver tissue damage and increases the probability of chronic liver disease.^93,94^ In general, *in vitro* inflammatory models are induced by exposing cells to inflammation, inducing substances, such as lipopolysaccharides (LPS), which can induce mild to severe inflammation depending on the concentration.^94^

**FIG. 5.**
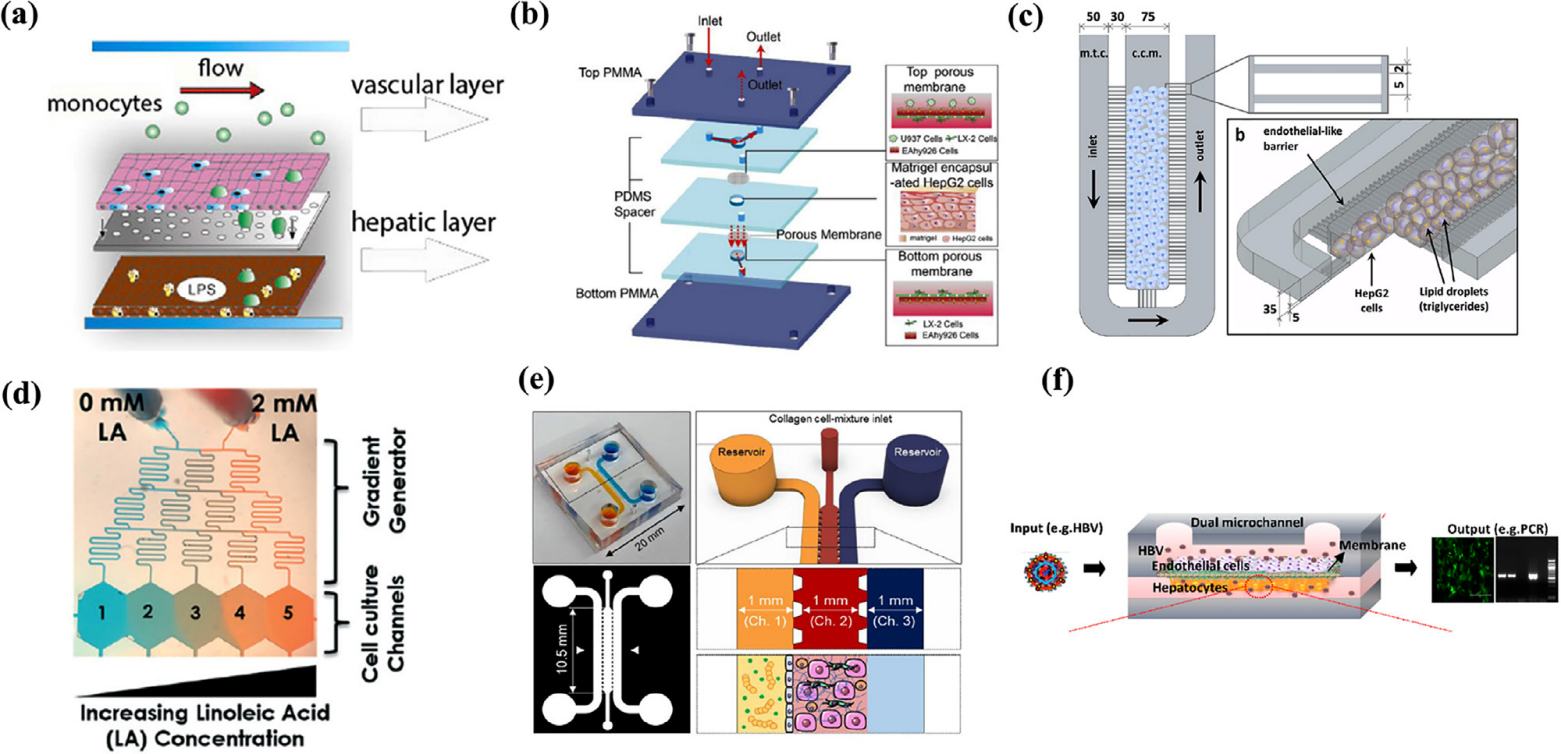
Disease models using liver-on-a-chip: (a) inflammation was induced by using TLR-stimulation on the vascular organoid liver chip. Reproduced with permission from Gröger *et al.*, Sci. Rep. **6**, 21868 (2016); licensed under a Creative Commons Attribution (CC BY) license.^97^ (b) ALD was induced by perfusing a medium containing ethanol to a chip to which four types of hepatocytes were applied. Reprinted with permission from Deng *et al.*, Biomed. Microdevices **21**, 57 (2019). Copyright 2019 Springer Nature.^104^ (c) Steatosis was induced by treating free fatty acid in the chip where the liver sinusoid was structurally implemented. Gori *et al.*, PLoS One **11**, e0159729 (2016). Copyright 2016 Author(s), licensed under a Creative Commons Attribution (CC BY) license.^107^ (d) NAFLD was induced according to the concentration gradient of linoleic acid in the concentration gradient generating chip designed in a tree shape. Reproduced with permission from Bulutoglu *et al.*, Lab Chip **19**, 3022 (2019). Copyright 2019 The Royal Society of Chemistry.^112^ (e) NASH was induced by exposing the liver-on-a-chip to FFA and LPS. Reproduced with permission from Freag *et al.*, Hepatol. Commun. **5**, 217 (2021). Copyright 2021 John Wiley and Sons.^113^ (f) HBV was induced in liver-on-a-chip by transfection with HBV-genome cDNA and virus genome expressed from recombinant adenovirus. Reproduced with permission from Kang *et al.*, Biotechnol. Bioeng. **112**, 2571 (2015). Copyright 2015 John Wiley and Sons.^119^

It is known that the immune response in the liver is mainly caused by non-parenchymal cells.^95^ In addition, there are research results that macrophage is a key factor in liver inflammatory response.^96^ A study by the Gröger group developed a microchip MOTiF biochip vascular organic-organoid model and demonstrated that toll-like receptor (TLR) agonists can be used to cause inflammation [Fig. 5(a)].^97^ The chip mimicked sinusoid while culturing human umbilical vein endothelial cell (HUVEC), monocyte, HepaRG, and stellate cells. Then, the authors observed that TLR stimulation induces the release of inflammatory/anti-inflammatory cytokines, reducing the expression of VE-cadherin and ZO-1 in endothelial cells and damaging the barrier. Gröger group conducted a follow-up study on the hypothermic storage of the chip.^98^ After storing the chip at low temperature for two days, their chip was treated with LPS to induce inflammation and an inflammatory reaction was observed.

LPS induces neutrophil accumulation in the liver and neutrophils adhere onto and crawl along LSEC monolayer during the recruitment.^99^ Long group developed an *in vitro* liver sinusoid chip by integrating the four types of primary murine hepatic cells into two adjacent fluid channels separated by a porous permeable membrane, replicating the liver's key structures and configurations.^55^ Authors treated the chip with LPS to induce inflammation. It was confirmed that neutrophils were accumulated in the chip as an inflammatory response to LPS. This work demonstrates that microfluidic liver-on-a-chip can be a useful *in vitro* platform for simulating and studying the dynamic immune responses involving multiple components, such as the blood vessel, immune cells, cytokines, hepatocytes, and other non-parenchymal cells.

### B. The fatty liver disease

The normal liver has the percentage of fat at around 5%. The fatty liver is a condition in which an excessive amount of fat is accumulated in the liver cells.^100^ The fatty liver is largely classified into alcoholic fatty liver and nonalcoholic fatty liver. Alcoholic fatty liver is caused by excessive alcohol consumption, and nonalcoholic fatty lipid disease (NAFLD) is caused by accumulation of triglycerides in the liver by obesity, regardless of alcohol consumption.^101^ Alcohol is one of the leading causes of liver disease, and alcoholic liver disease (ALD) accounts for 10% of all disease deaths worldwide. In addition, ALD is associated with more than 60 diseases, including hepatitis, cirrhosis, and insulin resistance.^102^ For this reason, the study of alcoholic fatty liver is important, but the mechanism for this is not fully elucidated, so the development of an *in vitro* model for this study is important.

It is known that the interaction between hepatocyte and non-parenchymal cells plays an important role in the progression of the fatty liver. Lee *et al.* designed a spheroid-based microfluidic system that included non-parenchymal cells to develop a 3D ALD model.^103^ The authors evaluated the changes in hepatocyte function by co-culturing rat primary cells and stellate cells on the chip and confirmed the recovery ability of liver tissue damaged by ethanol for 48 h. It was observed that as the concentration of ethanol exposed to the cells increased, the roughness of the spheroid surface increased, and the cell viability decreased. In addition, albumin and urea secretion were measured to evaluate the reversibility of hepatocyte function impaired by ALD. It was confirmed that reversible ALD damage occurred at an ethanol concentration of 60 *μ*l/ml and irreversible ALD damage at 80 *μ*l/ml. Because liver damage induces proliferation of stellate cells and secretion of ECM protein, the authors also examined the activity of stellate cells after ethanol treatment. This system successfully demonstrates *in vitro* reproduction of ALD and its applicability to ALD therapeutic drug screening.

Non-parenchymal cells play a key role in the complex process of ALD. Lin *et al.* induced ALD by perfusing a medium containing ethanol to the chip in which four cells were applied [Fig. 5(b)].^104^ In this system, hepatocytes, Kupffer cells, endothelial cells, and stellate cells were co-cultured to implement liver sinusoids to mimic the liver more physiologically, and it was confirmed that liver function was improved by measuring albumin secretion and urea synthesis. The authors identified markers at various concentrations of ethanol exposed to cells in this system. As a result of radical oxygen species (ROS) production, which plays an important role in the process of ALD and causes cell death and DNA damage, it was confirmed that it increased with the concentration and exposure time of alcohol. In addition, as the alcohol concentration increased, the expression of VE-cadherin, a tight junction marker of vascular endothelial cells, decreased. When authors measured the expression of endothelial nitric oxide synthase (eNOS), which induces the production of nitric oxide, it was observed that it significantly decreased with the concentration and time of alcohol exposed. On the other hand, it was observed that the expression level of alpha-SMA, a vascular endothelial cell growth factor, was increased, and it was confirmed that liver fibrosis was induced through this process.

NAFLD is the most common chronic liver disease worldwide and is a disease leading to cirrhosis and liver cancer, and also associated with type 2 diabetes.^105^ Since liver cancer is one of the top three causes of death in the world, early diagnosis of NAFLD is very important.^106^ However, the development of an *in vitro* model for this study is important because the mechanism of NAFLD has not been fully elucidated.

The sinusoid structure of the liver affects the blood flow, and the resulting concentration gradient of substances can affect the physiological function of cells. In a study by Rainer *et al.*, palmitic acid and oleic acid, free fatty acids (FFA), were applied to induce steatosis in the chip where the liver sinusoid is structurally implemented. The accumulation of triglycerides occurred at a slower rate in the chip compared to the 2D well plate culture. This is possibly a closer implementation of the state of chronic steatosis observed *in vivo* than conventional *in vitro* models [Fig. 5(c)].^107^

The 3D structure of the liver tissue can also play an important role. In a study by Hughes *et al.*, NAFLD was implemented using LiverChip^®^, which can cultivate hepatocytes in a collagen scaffold in 3D form.^108^ Cells were exposed to FFA to induce steatosis, and fat reduction was confirmed using therapeutic agents, such as pioglitazone and metformin. Through this study, the authors confirmed the possibility that a chip-based disease model could be used to measure the effects of various drugs on the progression or prevention of NAFLD.

In a study by Sung *et al.*, authors created an *in vitro* model of initial inflammation (NASH) to fibrosis of NAFLD on a chip capable of culturing hepatocytes and endothelial cells in 3D form using gelatin hydrogel.^109^ The authors observed that hepatocytes and endothelial cells cultured in 3D form were properly differentiated. Palmitic acid was added to the medium to induce NAFLD, and fibrosis was induced by adding TGF-beta to the NASH-inducing medium. TGF-beta is a protein that induces inflammation and fibrosis in stellate cells.^110^ It was confirmed that steatosis was reduced by treatment with Ezetimibe, which is known as a therapeutic for the fatty liver disease, to prove that it can work as a disease model capable of screening for drugs.

The Usta group created a patterned NAFLD model on the chip using a free fatty acid gradient to reproduce the spectrum of the disease states in a single continuous liver tissue.^111^ The authors generated a concentration gradient of fatty acid in the chip and evaluated the amount of fat accumulation. It was confirmed that the higher the fatty acid concentration, the higher the fat accumulation and the expression of lipid metabolism markers. Insufficient oxygen supply is known to be associated with the induction of NAFLD. It was confirmed that the fat accumulation amount increased with decreasing oxygen concentration [Fig. 5(d)].^112^ The NAFLD is characterized by a gradual increase in lipid accumulation in hepatocytes, which in turn leads to fibrosis and inflammation, and can ultimately lead to cirrhosis and liver cancer. In this study, it is meaningful that the range of disease progression and associating characteristics of this disease were reproduced by using the concentration gradient of fatty acids and oxygen.

In a study by Janget al., an *in vitro* model of NASH was developed using chips co-cultured with four cells: hepatocyte, Kupffer cell, liver sinusoidal endothelial cell, and hepatic stellate cell [Fig. 5(e)].^113^ Four types of primary cells were co-cultured with stable viability, and it was confirmed that liver-specific albumin and urea secretion function was improved. NASH was induced by exposure to FFA and LPS, and expression levels were observed by analyzing inflammatory markers MCP1, TNF-alpha, TGF-beta, and OPN. It was confirmed that liver damage was alleviated by applying elafibranor, a therapeutic agent, to confirm the possibility of applying the therapeutic agent screening. This study is unique that it has 3D microfluidic culture system that captures the essential morphologic features of NAFL and NASH and the progression from NAFL to NASH.

The adsorption of hydrophobic compound onto the surface of PDMS is a well-known problem.^114^ This issue can be particularly problematic in the case of NAFLD models, since hydrophobic compounds are involved. Kamei *et al.* developed a NAFLD model using a chip made of cyclo-olefin polymer (COP).^115^ Hepatocytes were cultured on a chip made of PDMS and a chip made of COP, and the adsorption of AdipoRed lipid dye was compared. It was observed that the adsorption of lipid dyes was lower in the COP material. Therefore, it is expected that a microfluidic chip made of a COP material with a weak degree of adsorption can be useful for future studies.

### C. Hepatitis B (HBV)

More than 240 million people worldwide are infected with HBV.^116^ In addition, HBV is a major health problem because it is one of the major causes of cirrhosis and liver cancer. However, it is difficult to detect this infection due to the loss of hepatocyte differentiation and phenotypic changes after a short period of time.^117^

In general, *in vitro* models of HBV are created by exposing liver cells to patient-derived HBV. Early chip-based HBV virus replication studies were performed by Noh *et al.* HBV was induced by transfection with HBV-genome cDNA on a microfluidic platform and by infection with a virus genome expressed from a recombinant adenovirus.^118^ The transfection method had a high infection efficiency for HepG2 cells, and in the case of primary rat hepatocytes (PRH), the adenovirus infection had a higher efficiency.

HBV also interacts with non-parenchymal cells like other liver diseases. Kang *et al.* induced HBV with recombinant adenovirus in a multicellular environment [Fig. 5(f)].^119,120^ In addition, it was confirmed that co-culture of endothelial cells consistently sustains albumin/urea secretion within the chip, and long-term studies that were previously impossible due to the rapid loss of function of hepatocytes in the existing cell culture system were possible.

In the study by Dorner *et al.*, HBV-on-a-chip was developed using LiverChip (CNBio), which can be cultured in 3D scaffold.^121^ It has been suggested that the 3D chip culture enhances the expression of innate immune responses in hepatocytes and enables the study of liver mechanisms. Kupffer cells did not respond to HBV infection initially, but it was confirmed that HBV infection was induced after the second stimulation by LPS. This platform is expected to be a novel *in vitro* tool for liver disease, liver physiology, and drug screening, which enables recreating the physiological structure of liver cells.

### D. Liver disease model using multiple organ chips

The intestine is the largest immune organ in the body, and the liver accounts for more than 70% of macrophages in the body.^122^ Therefore, an *in vitro* model of liver diseases in the context of the gut–liver axis is important.

In the study by Lauffenburger *et al.*, a microfluidic system was developed that allows intestinal cells and hepatocytes to be co-cultured on a chip.^123^ Intestinal cell barrier integrity and intestinal mucus and albumin production were measured to confirm intestinal and hepatic functions for 15 days. In addition, LPS was added to the circulating medium to induce inflammation in the intestinal cells and hepatocytes. As a result, it was observed that the expression of the inflammatory cytokines IFN-α, IFN-β, and IFN-γ was increased.

In fact, a fatty liver is formed by complex mechanism of action, as fat components introduced into the body through the oral cavity pass through the intestinal barrier, enters the systemic circulation before accumulating in the liver tissue. During the process, several biotransformation process is also involved.^124^ However, in a typical fatty liver chip model, only liver cells are cultured, and it is difficult to reproduce the process in which fat components are absorbed in the intestine and accumulate in the liver.

In a study by Sung *et al.*, a NAFLD model was developed using intestinal-liver chips that can simulate intestinal absorption and liver metabolism.^125^ After co-culture of intestinal cells and hepatocytes on the chip, fatty acids were treated only in the intestinal layer, so that the fat components were absorbed in the intestine and accumulated in the liver. In addition, the effect of butyrate and α-lipoic acid (ALA), which are substances that are known to inhibit fatty liver, and tumor necrosis factor-α (TNF-α), which is a substance known to promote the fatty liver disease, was examined. These compounds exerted effects that are consistent with previously known mechanisms of action. In a follow-up study, fatty acid absorption was evaluated under various culture conditions, and the anti-lipidemia effects of turofexorate isopropyl (XL-335) and metformin, candidate drugs for NAFLD, were confirmed.^126^

## V. REMAINING CHALLENGES AND CONCLUSION

Although liver‐on‐a‐chip systems have shown considerable potential, there are several challenges to be overcome in the development of more improved *in vitro* models. First, human-originated cells with improved physiological relevance should be applied to the liver-on-a-chip system to reproduce the function of the liver more accurately. Several studies have used immortalized cell lines, such as HepG2 cells, because the cell lines are cheap, stable, and cellular features can be easily controlled. However, these immortalized cells originate from cancer, and their functionality is limited. To overcome these limitations, primary hepatocytes have been used for the construction of liver-on-a-chip in recent studies. Although primary cells have exhibited better functions, the application of primary cells poses hurdles owing to difficulty in obtaining from humans and maintenance of functions. Alternatively, hiPSCs can be used for the development of the liver-on-a-chip system. Although hiPSCs cannot be easily differentiated into liver cells, they can provide personalized biological information with physiological relevance to humans. Moreover, the hiPSC-based organoid model can simulate liver function and help in the development of accurate treatment approaches for liver diseases in patients.

Second, biomechanical stimulation is thought to better reproduce the liver function and pathophysiology. Biomechanical stimuli include passive and active stimuli. Passive stimuli comprise stiffness, topology, and structural confinement, and active stimuli include compression, stretch, and shear stress.^127^ To reproduce liver, shear stress was applied using flow-based dynamic cultures that resulted in improved hepatic functions. Moreover, the sinusoidal topology and structure were recapitulated by hydrogel-based layer-by-layer co-cultures in the liver-on-a-chip. However, few studies have considered the stiffness of the ECM in the liver-on-a-chip system. The healthy liver matrix has a stiffness of 150 Pa. The stiffness increases to 1–6 kPa during the progression to fibrotic liver.^128^ Therefore, the effect of stiffness on liver cells in liver-on-a-chip model should be studied to mimic the pathophysiology of liver diseases.

Third, a deeper understanding of the interaction with the immune system is necessary for elucidating the pathogenetic mechanisms of liver diseases. Several research groups have studied multi-organ models, such as the gut–liver, liver–pancreas, and liver–kidney models.^129,130^ However, detailed aspects of inflammation and its role in the pathogenetic mechanism of disease development have not been reproduced correctly. Drugs for complex diseases can be developed if an accurate model that recapitulates multi-organ and immune system interactions is available. In a study by Sasserath *et al.*, multi-organ, pumpless immune system-on-a-chip was developed. THP-1 immune cells, cardiomyocytes, skeletal muscle cells, and primary hepatocytes were co-cultured in separate compartments. They demonstrated that this model can mimic both the targeted immune response and the general inflammatory immune response.^131^

Finally, as the liver performs diverse functions, often via interaction with other organs, the maintenance of its full functions is important in liver-on-a-chip systems. To this end, the secreted metabolites and metabolized drugs should be continuously collected from the chip for the analysis of liver functions. However, the repeated collection of samples intervenes with chip operation and affects the concentration of samples. Therefore, real-time monitoring systems should be integrated into the chip. In a similar perspective, combination of a miniaturized microscope with a chip can provide real-time information on the cellular morphology. There are many readouts that can be done on chip. Examples include immunohistochemistry, permeability, trans epithelial electric resistance (TEER), migration assays, angiogenesis, and other assays.^132^

In this review article, we have introduced various liver-on-a-chip systems for the study of drug metabolism and liver diseases. Microtechnology allows the improvement in conventional *in vitro* systems, and more physiologically relevant liver models have been developed recently. Furthermore, the liver system was connected with intestinal models through microfluidics and helped to mimic the absorption and metabolism of drugs. Liver-on-a-chip models have been used to mimic the characteristics of liver diseases to understand the pathophysiology and for the development of drugs. The liver is a central organ of the human body functions and metabolism. In the near future, the liver-on-a-chip system may be a key module for studying multi-organ interactions and for predicting the systemic response to drugs. The studies reviewed in this article show the potential of the liver-on-a-chip system and highlight the direction of progress with the application of the models.

## Data Availability

Data sharing is not applicable to this article as no new data were created or analyzed in this study.
